# Quantifying circulating antibody activities against the emerging environmental pathogen, *Streptococcus agalactiae,* in wild captured bull sharks, spotted eagle rays, bottlenose dolphins, and loggerhead turtles

**DOI:** 10.1016/j.fsirep.2021.100024

**Published:** 2021-09-02

**Authors:** Kursten A. Anderson, Adam M. Schaefer, Charles D. Rice

**Affiliations:** a Department of Biological Sciences, Graduate Program in Environmental Toxicology, Clemson University, Clemson SC 29634, USA; b Harbor Branch Oceanographic Institute at Florida Atlantic University, Ft. Pierce, FL 32963, USA; c Abt Associates, Atlanta, GA 30345, USA

**Keywords:** Bull shark IgM, Spotted eagle ray IgM, Bottlenose dolphin IgG, Loggerhead turtle IgY, *Streptococcus agalactiae*, mAb, Oceans human health

## Abstract

Global *Streptococcus agalactiae* and related pathogen infections are increasing, and place both wildlife species and humans at risk. Notably, *Streptococcus agalactiae* is now considered to be an emerging and zoonotic pathogen. Mouse monoclonal antibodies (mAbs) were generated against bull shark, *Carcharhinus leucas*, and spotted eagle ray, *Aetobatus narinari*, IgM, and bottlenose dolphin, *Tursiops truncatus*, IgG. Along with a previously generated mAb against loggerhead, *Caretta caretta*, IgY, these biological reagents were used to compare the sensitivity of relative antibody titers at sample dilutions of 1:200 in buffer to calculated antibody activities against whole cell *Streptococcus agalactiae* in wild captured individuals. Robust antibody responses were observed in each species, though some individuals differed significantly depending on whether viewed as relative antibody titers at sample dilutions of 1:200 or as antibody activities. The data from this study indicate that calculated antibody activities are more informative than relative antibody titers in determining antibody responses despite being more labor-intensive, expensive, and time consuming. This study provides several novel antibody reagents to the greater community of comparative immunologists, with a particular application to monitoring sentinel species for exposure to new emerging and zoonotic pathogens.

## Highlights

• *Streptococcus agalactiae* is an emerging environmental pathogen in the aquatic environment.
• Monoclonal antibodies were generated against bottlenose dolphin IgG and against spotted eagle ray and bull shark IgG.
• A previously generated monoclonal antibody against loggerhead sea turtle IgY was used to compare marine vertebrate responses.
• Relative antibody titers against *S. agalactiae* were quantified and compared to calculated antibody activities.
• Calculated antibody activities are more sensitive than simply relative antibody titer data.

In this study our goal was to develop monoclonal antibodies (mAbs) against bull shark, *Carcharhinus leucas*, and spotted eagle ray, *Aetobatus narinari*, immunoglobulin (Ig) M (IgM), bottlenose dolphin, *Tursiops truncatus*, IgG, and, along with a previously described mAb against loggerhead sea turtle IgY, compare relative antibody titers to activities against an aquatic strain of *Streptococcus agalactiae*, an emerging environmental pathogen [1], [2], [3]. *Streptococcus agalactiae* (group B streptococcus) is a Gram-positive bacterial pathogen known to cause septicemia, necrotizing fasciitis, and large mortality events in a variety of aquatic organisms. For example, this bacterium is known to affect crocodiles, frogs, and an array of bony fish species [4], [5], [6], [7], [8], [9] and elasmobranchs [10,11]. In humans and bovines, it is known to cause neonatal meningitis and mastitis, respectively [12,13]. *Streptococcus agalactiae* has also been isolated from both captive and wild bottlenose dolphins [1,14,15]. Due to its ability to adapt and survive in a wide variety of environments, more outbreaks of *Streptococcus agalactiae* are likely to occur in the aquatic environment as the main route of exposure is from the ingestion of streptococcal contaminated materials [1,16,17]. In addition, outbreaks could have implications for humans and aquatic species as some studies suggest that disease associated with this pathogen may be caused by the same or similar strains of *S. agalactiae* [1,9,18,19].

Bull Sharks (n = 10) and Spotted Eagle Rays (n = 8) were captured as part of a fishery-independent survey in the Indian River Lagoon (IRL) between Brevard and Martin Counties of Florida USA between 2017 and 2019 [20]. A blood sample (<1% of body weight) was collected from each individual via caudal vein or wing vein (rays) puncture with an 18 or 20 gauge 1-1.5-inch needle connected to a 10 ml syringe and immediately transferred to a serum separator tube. Blood samples were centrifuged for 10 min at 1000 x *g*. Serum was then transferred to cryovials and stored at -80°C prior to shipping to Clemson University facilities. Bottlenose dolphin serum samples (n = 33) were randomly chosen from limited samples stored and used in previous studies [21], [22], [23], [24], [25], [26]. An in-hand monoclonal antibody (mAb LH-9) against loggerhead sea turtle IgY has been previously described with technical applications [27,28]. Serum samples (n = 9) from loggerhead turtles were randomly chosen from limited samples stored and previously used in other studies [29].

**Fig. 1:**
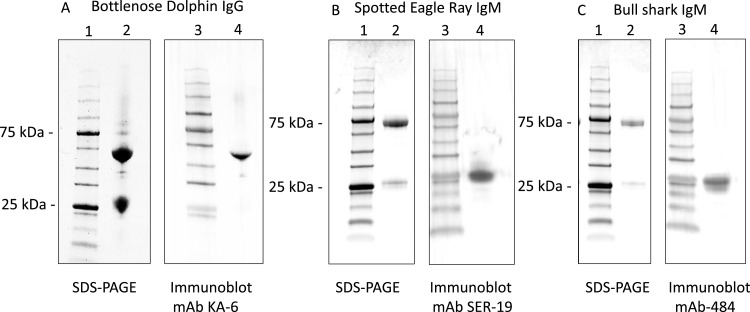
(A) SDS-PAGE and Coomassie blue staining of purified bottlenose dolphin IgG products, followed by Western blotting with mAb KA-6. Lane 1: molecular weight marker; Lane 2: purified IgG; lane 3; molecular weight standard; lane 4 immunoblot results showing recognition of 55 kDa heavy chain. (B) SDS-PAGE and Coomassie blue staining of purified spotted eagle ray Ig products, followed by Western blotting with mAb SER 12-9. Lane 1: molecular weight marker; Lane 2: purified Ig; lane 3; molecular weight standard; lane 4 immunoblot results showing recognition of 27 kDa product. (C) SDS-PAGE and Coomassie blue staining of purified bull shark Ig products, followed by Western blotting with mAb 484. Lane 1: molecular weight marker; Lane 2: purified Ig; lane 3; molecular weight standard; lane 4 immunoblot results showing recognition of 27 kDa product.

Procedures to purify IgM from bull shark and spotted eagle ray serum samples using Protein-A columns were previously described for the Atlantic sharpnose shark, *Rhizoprionodon terraenovae*, [30]. This approach was previously shown to be efficient for isolation of IgM from several species of fish, including elasmobranchs [30], [31], [32]. Due to the loss of previously generated hybridomas secreting mAbs against bottlenose dolphin IgG, it was necessary to generate another source using previously reported approaches [25]. Mouse mAbs were generated using routine procedures as described in previous studies from our lab [33,34]. Subsequent testing revealed that mAbs SER12-9, 4F8, and KA-6 were most suitable for spotted eagle ray, bull shark, and bottlenose dolphin immunoglobulin (Ig), respectively. Each of these three mAbs were found to be an IgG_1_ κ isotype. Purified Ig products from the two elasmobranchs and bottlenose dolphin were then subjected to SDS-PAGE under reducing conditions and immunoblotting using species-specific mAbs to verify specificity against targeted proteins of interest. We found that purified bottlenose IgG consists of the expected ≈ 55 kDa heavy chains and ≈ 25 kDa light chains (Fig. 1A). Under similar conditions purified spotted eagle ray and bull shark Ig consists of expected ≈ 75 -78 kDa heavy chains and ≈ 27 kDa light chains of IgM (Figure B, C). It is noted that the smaller Ig product is less abundant than the heavier Ig product for both elasmobranchs. This outcome was previously observed while purifying serum IgM from Atlantic sharpnose sharks wherein the heavier and lighter purification products were not equally abundant [30]. Such discrepancy in relative amount of heavy and light products of IgM upon protein-A purifications can also observed in bony fishes, depending on the species [31]. However, it is possible that we co-purified small amounts of IgNAR that may have degraded from the expected product [35,36]. Since IgNAR chains are not associated with light chains our heavier chains would be more brightly stained if in fact we co-purified small amounts of IgNAR. To explore these questions more thoroughly, future studies should immunoprecipitate whole serum proteins from spotted eagle rays and bull sharks with mAbs SER12-9 and 4F8, respectively, as described by Smith et al [37]. Our monoclonal antibody KA-6 is specific for the heavy chain of bottlenose dolphin IgG, and mAb SER12-9 and mAb 484 are specific to what appear to be the light chains of spotted eagle ray and bull shark IgM, respectively, though the light chains of IgW cannot be ruled out at this time. Also, we do not know at this time which light chain isotype(s) are recognized by mAbs SER12-9 and 484. Nonetheless, we have mAb reagents against purified Ig products that are specific to the lighter of two protein products revealed under reducing conditions (Fig. 1 B, C).

*Streptococcus agalactiae* cultures from an aquatic strain were a generous gift from Dr. John Hawke, Louisiana State University, and grown as directed [9]. High bonding 96 well plates (Corning, #9018) were coated for 16 h at 4°C with 75 µl of a 1 mg ml^−1^ solution of poly-D-lysine (VWR Scientific) in distilled water, followed by wash steps with phosphate buffered saline (0.01 M, pH 7.2) containing 0.05% tween-20 (PBS-TW20). To perform ELISAs against whole cell *Streptococcus agalactiae* cultures adhered to ELISA plates, cultures were first grown under conditions as previously described [29]. Optimized suspensions of live bacterium were then prepared and 50 µl of bacterium were added to wells using our lab's modifications [25,29,30] of the original developers of the assay [38], including fixation to the plates and subsequent blocking steps. To initiate assays, serum suspensions were made by adding 10 µl of serum from each individual animal into 990 µl of PBS-TW20 in 1.5 ml snap cap tubes to yield a dilution at 1:100. As a reference sample, a composite sample for each species comprised of serum from randomly selected individuals in PBS was made at a dilution of 1:100. Eighty µl of each diluted sample, including the composite reference sample, were added in duplicate to the respective wells and serially diluted to give 1:100, 1:200, 1:400, 1:800, and 1:1600 dilutions, followed by the addition of 40 µL PBS-TW20 to yield final dilutions of 1:200, 1:400, 1:800, 1:1600, and 1:3200. Each plate also contained two wells of 80 ul PBS-TW20 as an assay control. The plates were incubated overnight at 4°C and washed 4 times with PBS-TW.

As the source of mAb specific to each species, hybridoma supernatants from single batch confluent cultures were diluted 1:3 with hybridoma media containing 10% FBS [33] and 80 µl added to all wells of the plate and incubated at room temperature for 2 hr, followed by fours washes in PBS-TW. The remaining steps were routine and described elsewhere [29]. Goat anti-mouse IgG AP (1:2000) was added in 75 µL volumes to each well and incubated at room temperature for 1.5 h before being washed four times with PBS-TW20. Finally, 80 µL of 1 mg/mL *p-*nitrophenol phosphate (ThermoFisher) in AP buffer were added to each well, the plates were incubated for 30 min at room temperature, and the reaction was stopped with 100 ul 1M NaOH. Optical densities for all plates were read at 405 nm and the data recorded. Relative antibody titers were calculated as the optical density (O.D.) multiplied by the serum dilution factor of 1:200 [22,26,29,30]. Antibody activity for each sample was determined using previously published methods [39,40] and expressed as units of activity per µL serum. In brief, dilutions were expressed as the equivalent volume of the undiluted serum determined by taking the product of the volume of dilution used (75 ul) and each of the dilution factors. The standard curve was then used to determine volume of serum at the 50% point of the maximum O.D. obtained for the standard pooled serum, and this volume was then assumed to be the equivalent of one unit of antibody activity (1 Unit/volume of sample). Each sample was plotted against the 50% point of the maximum O.D. from the standard curve to determine the volume of test sample at that 50% point of the standard. To determine the activity for each of the test samples, the following equation was used: Activity of test sample = (activity of standard) x (50% volume of standard divided by 50% volume of test sample).

**Fig. 2:**
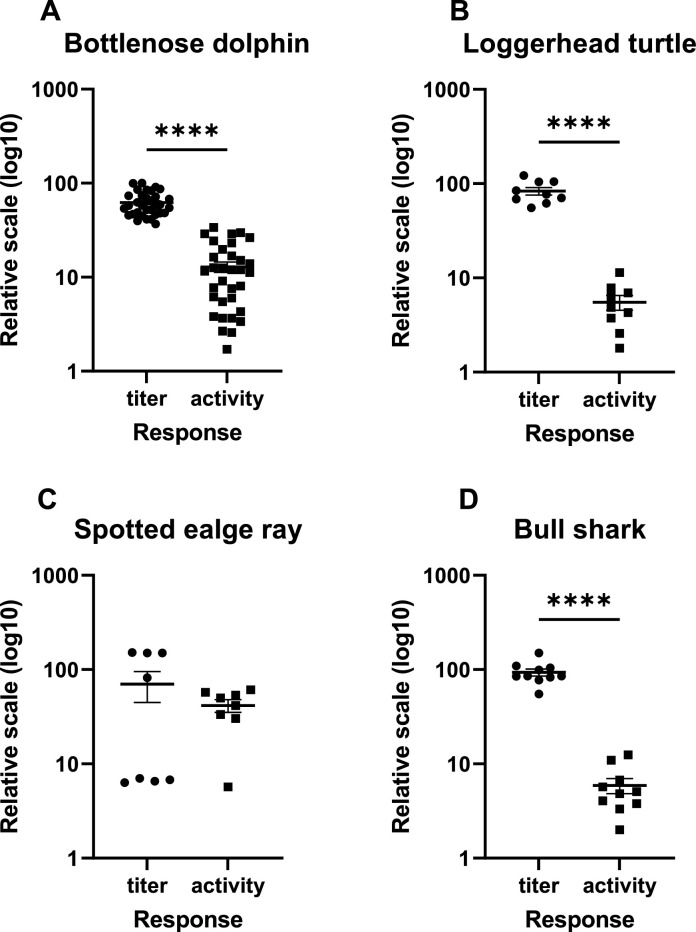
Comparison of relative antibody titers of serum or plasma dilutions at 1:200 and antibody activities (U/µl) for (A) bottlenose dolphins, n = 33, (B) loggerhead sea turtles, n = 9 (C) spotted eagle rays, n = 8 (D) bull sharks, n = 10. Significant differences between the mean relative titer and mean activities were found for bottlenose dolphins (A), loggerhead turtle (B), and bull shark (D). Spotted eagle relative titers and antibody activities did not differ (C). *** denotes *p*≤0.001; **** denotes *p<*0.0001.

For statistical analysis, relative antibody titers from each species at a 1:200 dilutions were compared to their calculated activities using a Mann-Whitney U test. Correlations between individual relative titers at 1:200 dilutions and the same individual's antibody activity were determined by Spearman's correlation coefficient using GraphPad Prism9’s statistical software. An α-value of 0.05 was established prior to the study. When comparing how relative antibody titers and antibody activities are calculated it is generally expected that titers would be statistically higher than activities, and this was the case except for spotted eagle ray samples (Fig. 2). In general, relative titers were tightly clustered in bottlenose dolphin, loggerhead, and bull shark samples, while in spotted eagle ray samples half had very low titers while the other half were roughly 10 times higher. Interpreting the significance of this is difficult because the sample size is very low (n = 8), and this is due to the sparse distribution of these animals during the time of field work. While not examined in this study, it is possible that some of the rays were previously exposed to *S. agalactiae* and their immunoglobulin responses were higher relative to the unexposed. As noted, antibody titers in bull shark are tightly clustered even though the sample size is similar (n = 10) to what was available for spotted eagle rays. In this case it may be that all bull sharks sampled were previously exposed to *S. agalactiae*, or that their Ig (IgM?) is cross-reactive with similar antigens. Also, if our mAbs are in fact specific to IgM light chains, we do not know if the ELISA assays are recognizing pentameric IgM, monomeric IgM, or both. There is much to explore and learn about how spotted eagle rays and bull sharks respond to environmental pathogens under carefully controlled conditions, and especially how these responses compare to elasmobranch species such as the nurse shark and spiny dogfish that have been examined more thoroughly [35,37,41].

Antibody activity data were much less clustered than relative titers. This variability is related to varying optical density values between samples as they are serially diluted, and thus some samples had higher signal levels at higher dilutions. In this view, it appears that antibody activity data are more sensitive and informative than relative titers in determining differences between samples. The highest available sample size was for bottlenose dolphins, and thus a large distribution of activities, ranging from 1.7 to 36 Units per μl sample (Fig. 2A). Activities in loggerhead samples ranged between 1.8 and 11.36 (Fig. 2B), between 5.7 and 57 for spotted eagle ray samples (Fig. 2C), and between 2 and 12.5 for bull shark samples (Fig. 2D).

**Fig. 3:**
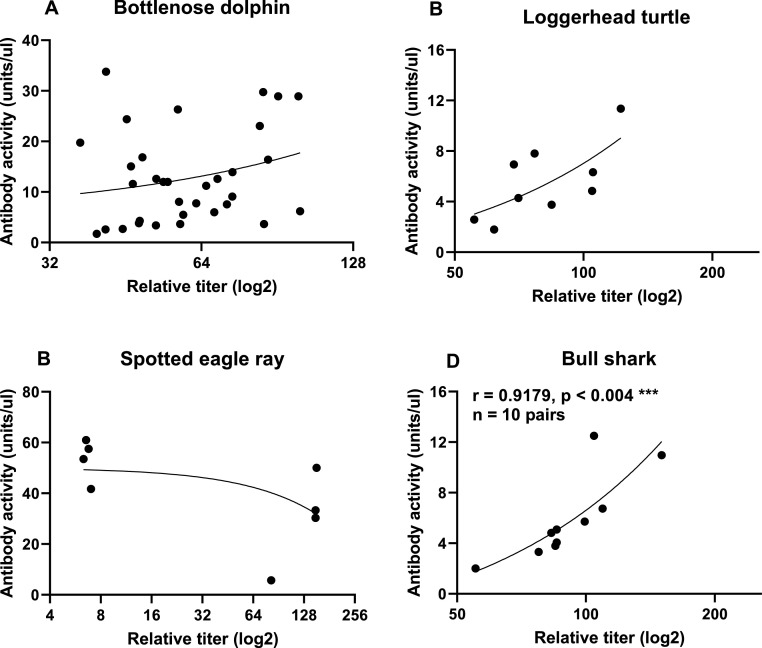
Spearman correlation analysis between relative titers of serum or plasma dilutions at 1:200 to antibody activities (U/μl) in (A) bottlenose dolphins, n = 33, (B) loggerhead sea turtle, n = 9, (C) spotted eagle rays, n = 8, (D) bull sharks, n = 10. No significant correlations were found for relative antibody titers and antibody activities in bottlenose dolphins, loggerhead sea turtles, and spotted eagle rays. There was a positive correlation between relative antibody titers and antibody activities in bull sharks (*** denotes *p* ≤ 0.004).

One of the questions asked at the onset of this study is can relative antibody titers predict antibody activity in environmental species. This is important because calculating antibody activities is much more labor intensive, requires significantly more research consumables, and is far more time consuming. Only bull shark antibody activities correlated with relative titers (Fig. 3). If sample sizes were larger for spotted eagle rays, bull sharks, and loggerhead turtles, there may be a better correlation. Why the discrepancy between relative titers and activities in spotted eagle ray samples and the other species is currently unclear at this time.

Secondary to the development of these reagents we demonstrate that calculating antibody activities is a more sensitive and possibly more informative approach than calculating simple relative titers for quantifying antibody responses to pathogens of interest. This may be especially important when considering that the type of antibody responses in lower vs higher vertebrates is different in both kinetics and antibody class [41,42]. Serum elasmobranch pentameric IgM contains natural antibodies against a variety of carbohydrates, glycosylated proteins, and pathogen associated molecular patterns, leading to a high degree of cross-reactivity [35]. Moreover, elasmobranchs secrete pentameric IgM early in specific immune responses and then monomeric IgM as the response matures [43]. Reptiles such as sea turtles secrete IgY as their mature antibody response to environmental pathogens [27,29,44], while marine mammals, like humans, generate and secrete IgG as the mature Ig response to pathogens [25,45,46]. Going forward it will be important to compare Ig-class responses against various pathogens in terms of antibody activities. As reagents become available for anti-IgM and Ig-other classes within the same species investigators can develop ELISA assays to monitor Ig class-specific antibody responses in a variety of aquatic vertebrates. Towards this end, our mAbs are now available to the larger scientific community interested in the immunology of bottlenose dolphins, loggerhead sea turtles, spotted eagle rays, and bull sharks. These gregarious marine organisms may have a significant role in the reciprocal interactions between aquatic species, humans, and aquatic pathogens from an Oceans and Humans (OHH) perspective, and this topic has been extensively reviewed [47,48]. The broader OHH community links the effects of multiple interactions between biotic and abiotic factors to understand interspecies environmental health [49], [50], [51], [52] . These environmental interactions also include the likelihood of humans coming into contact with aquatic pathogens associated with known and emerging environmental pathogens typically contained within wildlife species [53]. Our reagents will be especially useful for those interested in these animals as sentinel species within the larger context of OHH.

## Declaration of Competing Interest

We declare no conflicts of interest
